# Radiographic diagnosis of Pneumoconioses by AIR Pneumo‐trained physicians: Comparison with low‐dose thin‐slice computed tomography

**DOI:** 10.1002/1348-9585.12141

**Published:** 2020-07-27

**Authors:** Shoko Nogami, Naw Awn J‐P, Munenobu Nogami, Tomomi Matsui, Nlandu Roger Ngatu, Taro Tamura, Yukinori Kusaka, Harumi Itoh, Narufumi Suganuma

**Affiliations:** ^1^ Department of Environmental Medicine Kochi Medical School Kochi University Nankoku Japan; ^2^ Department of Radiology Kobe University Hospital Kobe Japan; ^3^ Department of Public Health Kagawa University Faculty of Medicine Kagawa Japan; ^4^ Fukui City Public Health Center Fukui Japan; ^5^ Department of Radiology Faculty of Medicine University of Fukui Japan

**Keywords:** chest radiography, computed tomography, pleural plaque, pneumoconiosis, sensitivity

## Abstract

**Objectives:**

The Asian Intensive Reader of Pneumoconiosis (AIR Pneumo) is a training program designed to improve diagnostic skills for chest radiographies (CXRs) in accordance with the ILO/ICRP 2000. The purpose was to determine the prevalence of occupational environmental pulmonary disease findings in construction workers on thin‐slice computed tomography (thin‐slice CT), and to compare the diagnostic performance with CXR evaluated by AIR Pneumo‐trained physicians.

**Methods:**

Ninety‐seven male construction workers underwent low‐dose thin‐slice CT and CXR on the same day. NIOSH B reader and a board‐certified radiologist each interpreted the thin‐slice CTs independently. The concordant findings on thin‐slice CT were established as the reference standard and were statistically compared with CXRs. Four physicians interpreted CXRs independently according to the ILO/ICRP 2000.

**Results:**

Of the 97 cases, nine showed irregular or linear opacities, and 44 had pleural plaques on thin‐slice CT. Five, four, three, and two of nine cases with irregular opacity were detected by the four readers on CXRs, respectively. Sixteen, 14, 9, and 5 of the 44 cases with pleural plaques were detected by the four readers, respectively. Specificities for irregular opacities ranged from 94% to 100%, and those for pleural plaques were from 86% to 96%.

**Conclusions:**

Thin‐slice CT‐detected irregular opacity was found in 9.3%, whereas pleural plaque was found in 45.4% among the construction workers. Chest radiography showed acceptable performance in classifying pneumoconiotic opacities according to ILO/ICRP 2000 by the AIR Pneumo and/or NIOSH‐certified physicians.

## 1 INTRODUCTION

Chest radiography is a standard tool used in medical screening for pneumoconiosis. In order to perform the medical screening and surveillance in a reproducible manner, the 2000 revised version of the ILO International Classification of Radiograph of Pneumoconioses (ILO/ICRP 2000)^1^ is applied internationally. However, a certain level of training is needed in order to understand and properly apply the classification system in daily clinical practice in an occupational health setting.

The Asian Intensive Reader of Pneumoconiosis (AIR Pneumo)^2^, ^3^, ^4^ was introduced as a training program for physicians to improve their diagnostic skills in the interpretation of chest radiographies in accordance with the ILO/ICRP 2000 and certifies physicians who pass an examination on reading and correctly classifying real cases of pneumoconioses. The AIR Pneumo certification examination is comparable to the United States National Institute of Occupational Safety and Health (NIOSH) B reader certification,^5^, ^6^ both of which evaluate a physician's proficiency in classifying chest radiography of pneumoconiosis with consistency.^2^

Chest computed tomography (CT) has been developed as a standard and robust imaging tool for lung disorders and has been confirmed as a benchmark to evaluate the detectability and diagnostic performance of the targeted imaging modalities of the lung. Especially for thin‐slice CT, its ability to characterize the early changes of pneumoconiosis, including morphological changes in the secondary pulmonary lobule,^7^, ^8^, ^9^, ^10^ is reportedly superior to the other modalities, yielding higher sensitivity for detection of early stage pneumoconiosis.^11^, ^12^, ^13^

Our hypothesis was that the AIR Pneumo program improves the diagnostic performance of chest radiography for occupational environmental pulmonary disease determined by the thin‐slice computed tomography (thin‐slice CT).

The purpose of the study was thus to determine the prevalence of findings for occupational environmental pulmonary disease in construction workers on thin‐slice CT. As medical screening by CXR is still important, we evaluated the diagnostic performance of chest radiography coded according to ILO/ICRP 2000 by AIR Pneumo‐ or NIOSH‐certified physicians using CT reading as reference.

## 2 MATERIALS AND METHODS

### 2.1 Subjects

The institutional review board approved this study. Informed consent for chest radiography and CT was acquired by all the enrolled subjects. Chest images taken for the low‐dose CT lung cancer screening program for a construction workers’ association performed at the University of Fukui were subjected to this study. From 2005 to 2006, 97 male construction workers (ages 49 to 77 years; average, 61.8 years) underwent low‐dose thin‐slice CT as well as chest radiography on the same day. A Japan Society for Occupational Health board‐certified occupational health physician with NIOSH B reader certification and a Japan Radiological Society board‐certified radiologist interpreted the thin‐slice CT independently using the international classification of thin‐slice CT for Occupational Environmental Respiratory Disease.^14^, ^15^, ^16^ The concordant findings between the two readers were established as the reference standard for thin‐slice CT findings and were compared with the findings on chest radiographies in the following statistical analysis.

### 2.2 Thin‐slice CT and chest radiography

A low‐dose CT scan was taken for each participant by 16‐channel multi‐detector CT (Sensation 16 Somatom, Siemens Healthcare) from the apex to the base of the lung with collimation of 2 mm, tube voltage of 120 kV, and tube current of 25 mA (radiation absorbed dose of less than 2 mGy) in a prone position with full‐inspiration breath‐hold. Image reconstruction was performed for thin‐slice CT images with a slice thickness of 2 mm using a high‐resolution algorithm (lung kernel) with reduced field of view targeting the ipsilateral lung and for thick‐section CT images with a slice thickness of 7 mm.

A posterior‐anterior projected digital chest x‐ray (CXDI, Canon Inc) was taken for each participant by flat‐panel detector using a high‐kilovoltage technique at 120 kV and 200 mA using a photo‐timer under breath holding at deep inspiration. The image was digitally printed on a medical quality laser printer.

### 2.3 Image analysis

All image analysis was performed for evaluation of parenchymal and pleural abnormalities.

Image interpretation of thin‐slice CT was performed by a physician in occupational health who specializes in occupational lung disease (15 years' experience) and a board‐certified radiologist (15 years' experience). Criteria for interpretation were based on the International Classification of high‐resolution (HR) CT for Occupational Environmental Respiratory Disease.^14^ Evaluation of parenchymal abnormalities was performed for findings of irregular or linear opacities in six regions in the chest (left and right sides; upper, middle, and lower lung fields, respectively) and scored using a 4‐point scoring system in each region. The scores were summed in each subject (Sum Grade, ranged 0 to 18) and were considered to be positive (abnormal) when the summed score was more than zero. For assessment of pleural abnormalities, pleural abnormalities are differentiated according to the CT appearance into parietal and visceral types according to the ICOERD. The term “parietal type” includes the typical tableland‐shape as well as less elevated thickening of the pleura without subpleural fibrosis. The existence of the findings of pleural plaque anywhere in the thorax was considered to be positive (abnormal), and their locations were recorded (parietal/visceral pleura, mediastinum, and diaphragm). We have carefully differentiated anatomical structure that mimic plaque, such as fat pad, muscles, and intercostal arteries.

Chest radiographies were interpreted by four physicians (A: NIOSH B reader, an occupational health physician with 15 years of experience; B: an AIR Pneumo‐trained occupational health physician with 6 years' experience of interpretation of chest radiography; C and D: AIR Pneumo‐trained physicians with 6 years' and 1 year's experience, respectively), each working independently according to the ILO International Classification of Radiograph of Pneumoconiosis (ILO/ICRP) 2000.^1^ Parenchymal abnormalities were evaluated based on the 12‐point scoring system and were defined to be positive (abnormal) when the score was equal to or higher than “0/1”. Usual cut‐off level of 1/0 was also employed for the analysis. The decision to use 0/1 as threshold rather than 1/0 follows a previous comparison study between chest radiographies and HRCT in pneumoconiosis.^17^ Pleural abnormalities were determined by the findings of pleural plaques or diffuse pleural thickening.

### 2.4 Statistical analysis

To acquire the reference standard for evaluation of diagnostic performance of chest radiography, prevalence (number of positive findings in respective patients evaluated by consensus reading of the two readers) was calculated on thin‐slice CT for parenchymal and pleural abnormalities, respectively.

To determine the diagnostic performance of chest radiography for irregular opacities and pleural thickenings, the findings of small opacities more than 0/1 and pleural plaques or diffuse pleural thickening on chest radiographies were statistically compared with the findings of irregular or linear opacities and pleural plaque on thin‐slice CT, respectively.

For patient‐basis analysis, sensitivities and specificities of the chest radiography were then calculated based on the findings of thin‐slice CT, followed by the McNemar's test to assess the statistically significant difference.

Finally, possible reasons and prevalence for false negative/positive cases on chest radiography were evaluated for irregular/linear opacities and/or pleural lesions determined by thin‐section CT as a reference standard.

## 3 RESULTS

Voluntary participants of 97 current or former carpenters belonging to the Carpenters’ Association underwent low‐dose CT screening for both evaluation of nonmalignant and malignant asbestos‐related diseases. In evaluation of parenchymal abnormalities, nine of 97 patients showed irregular or linear opacities (Table 1). Regarding pleural abnormalities, 44 patients had pleural plaques on thin‐slice CT. The other evaluated CT findings which were concordant between readers were as follows: ground glass opacity (n = 1), emphysema (n = 18), honeycombing (n = 1), suspect of lung cancer (n = 1), and pleural calcifications (n = 6). Parenchymal abnormalities coexist, for example, five cases with both irregular opacity and emphysema, one of which also had ground glass, and a case of combination of irregular and honeycombing. The rest of the findings related to the pneumoconiosis including rounded opacity, large opacity, or diffuse pleural abnormalities were not found in our study population.

**TABLE 1:** Low‐dose thin‐slice CT finding of 97 construction workers

Findings	Number (%)
Parenchyma	
Irregular opacities	9 (9.3%)
Ground glass opacities	1 (1.0%)
Emphysema	18 (18.6%)
Honeycombing	1 (1.0%)
Lung cancer	1 (1.0%)
Pleura	
Pleural plaque	44 (45.4%)
Pleural calcification	6 (6.2%)

Among these findings detected by CT, we focused on irregular opacities and pleural abnormalities that are compatible with asbestos‐related diseases (Table 2). At least one reader detected 66.6% (6/9) irregular opacities classified 0/1 or greater and 44.4% (4/9) of those classified 1/0 or greater on chest x‐ray. ILO/ICRP guided reading by AIR Pneumo‐ or NIOSH‐certified physicians showed specific results that four readers agreed negative in 90.9% (80/88) or 93.2% (82/88) when cut‐off level of 0/1 or 1/0 was applied, respectively. However, the sensitivity of detecting plaque on chest x‐ray was relatively low as 43.2% (19/45), and specificity for plaque detection was acceptably high as 78.8% (41/52).

**TABLE 2:** The proportion of chest x‐ray readings that agreed with CT‐proven gold standard among the 97 construction workers

Number of CXR readers	Number of detected cases in CXR (%)	
Irregular opacities		
Cut‐off level	0/1 or greater	1/0 or greater
Sensitivity (in 9 positive cases)		
4 readers	2 (22.2%)	2 (22.2%)
3 or more readers	3 (33.3%)	2 (22.2%)
2 or more readers	3 (33.3%)	3 (33.3%)
1 or more readers	6 (66.6%)	4 (44.4%)
Specificity (in 88 negative cases)		
4 readers	80 (90.9%)	82 (93.2%)
3 or more readers	88 (100%)	88 (100%)
Plaque		
Sensitivity (in 45 positive cases)		
4 readers	4 (9.1%)	
3 or more readers	7 (15.9%)	
2 or more readers	14 (31.8%)	
1 or more readers	19 (43.2%)	
Specificity (in 52 negative cases)		
4 readers	41 (77.4%)	
3 or more readers	49 (92.5%)	
2 or more readers	51 (96.2%)	
1 or more readers	52 (100%)	

Note

Chest x‐ray readings for irregular opacities were dichotomized at cut‐off level of 0/1 or 1/0.

**FIGURE 1:**
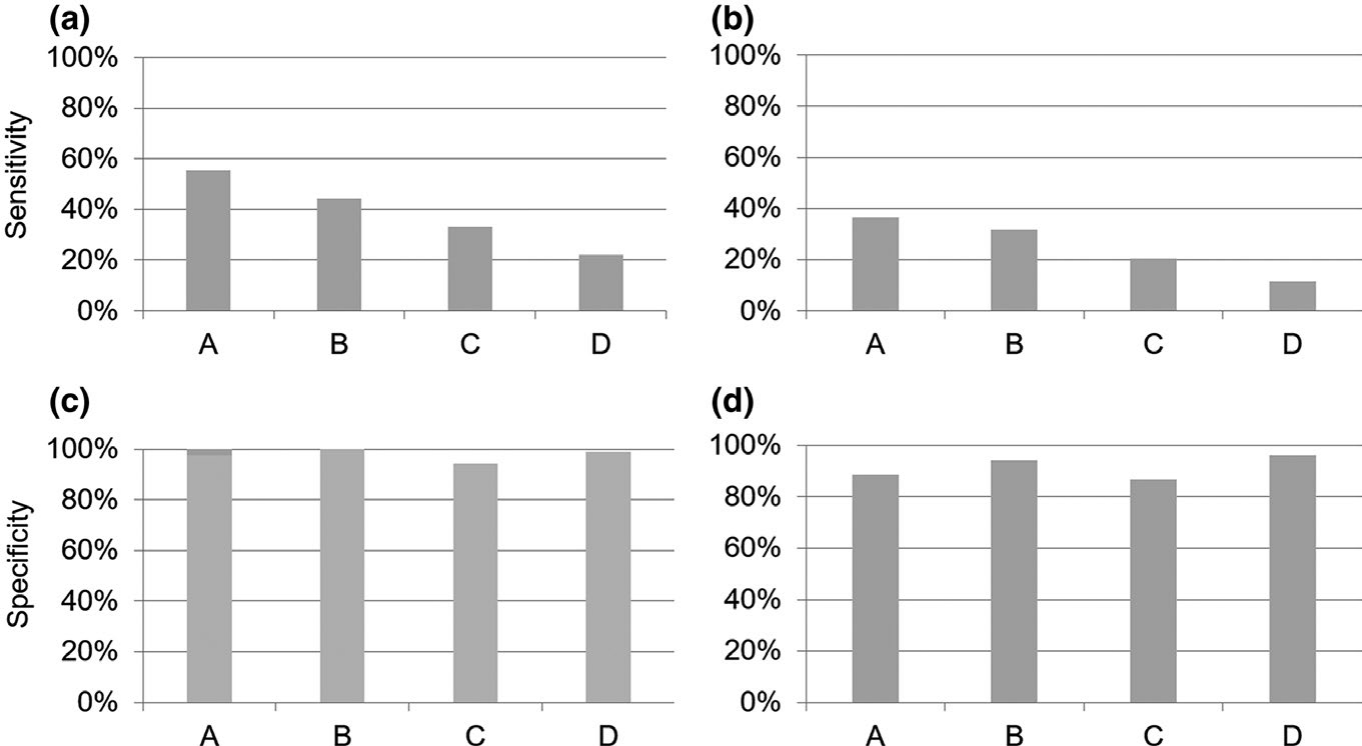
The diagnostic performance of the readers (A: NIOSH B reader, an occupational health physician; B: AIR Pneumo‐trained occupational health physician with 6 y of experience; C and D: AIR Pneumo‐trained physicians with 6 and 1 y of experience, respectively). (a) Sensitivity of detecting irregular opacities on chest radiography, (b) Sensitivity of detecting pleural plaques on chest radiography, (c) Specificity of detecting irregular opacities on chest radiography, and (d) Specificity of detecting pleural plaques on chest radiography, against thin‐slice CT as a reference standard

Of the nine cases with irregular opacity, five, four, three, and two small opacities were detected by readers A, B, C and D on chest radiographies, respectively (Figure 1). In reader A, three subjects with scores of 0/1 for irregular/linear opacities could be detected; however, the other readers were not able to detect those lesions. Sensitivity showed a positive trend toward the experience level of readers although a statistically significant difference could not be established due to the small number of positive patients. Specificities for irregular opacities ranged from 94% to 100%, which showed no statistically significant difference between readers (*P* > .05),

Of the 44 cases with pleural plaques, 16, 14, 9, and 5 pleural plaques were detected by each reader, respectively. Sensitivity of reader A (36.4%) was significantly higher than reader C (20.5%) and reader D (11.4%) (*P* = .016 and .010, respectively), and that of reader B (31.8%) was significantly higher than reader D (*P* = .039). Specificity for pleural plaques was from 86% to 96% and there was no statistically significant difference between readers.

As for the false positive cases on chest radiography, eight cases were misclassified as irregular opacity by one of four readers. Details of CT findings of these false positive cases are six cases with plaques, calcified plaques (n = 3), emphysema (n = 1), and negative case (n = 1). Pleural lesions were erroneously diagnosed as positive because of rib fracture (n = 1) and pleuritis (n = 4). Patients with small plaques and/or subtle irregular opacities were missed on chest radiography, resulting in false negative cases. Three cases with irregular opacity which three among four readers could not detect, and one case of irregular opacity with emphysema and pleural plaque could not be detected by two readers, especially the irregular opacity was detected by only one reader. Two cases with irregular opacity plus emphysema, of which just one reader detected irregular opacity in one of these two. These are cases with subtle irregular opacity, and with the presence of emphysema and/or plaque it becomes even harder to detect these findings on chest radiographies.

## 4 DISCUSSION

In our study, chest radiography, interpreted by trained AIR Pneumo or NIOSH B readers with various background, showed considerably high sensitivity and specificity in detecting nonmalignant asbestos‐related diseases when compared with thin‐slice CT results as a reference standard. Systematic description of parenchymal and pleural findings on the chest radiography according to ILO/ICRP guides readers to provide reproducible reading results.

In our study population, findings of severe pneumoconiosis including rounded opacity, large opacity, or diffuse pleural abnormalities were not observed on thin‐slice CT, which was consistent with the imaging findings for construction workers with mild exposure to asbestos and other dusts. Recent improvements in working conditions and changes in industrial structure have reduced the number of positive patients with pneumoconiosis as well as the number of new cases,^18^ resulting in a relatively large number of the patients with scores of less than 1/2 as compared with the severe cases with scores over 2/1^18^, ^19^ diagnosed in dust‐exposed workers in the past.^20^ This situation in turn emphasized the importance of diagnostic ability in interpreting subtle findings with pneumoconiosis on chest radiographies, which eventually leads to a need for a solid and robust educational system for physicians in charge of interpreting screening chest radiographies.

**FIGURE 2:**
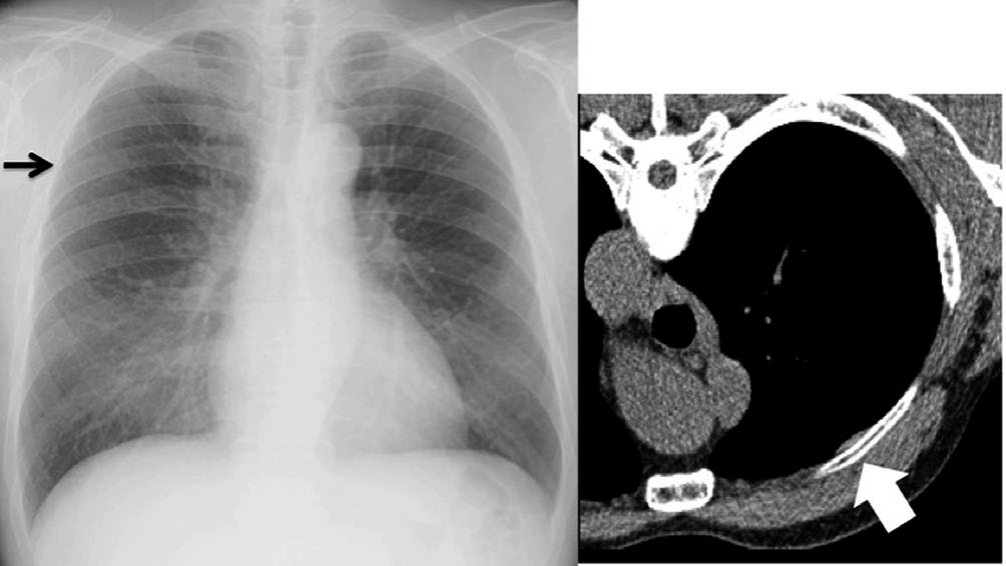
Discrepancy between chest radiography and thin‐slice CT reading results (Case 1). CT‐proven subtle pleural plaque (white arrow) was not detected on chest radiography (black arrow) by any of the readers

**FIGURE 3:**
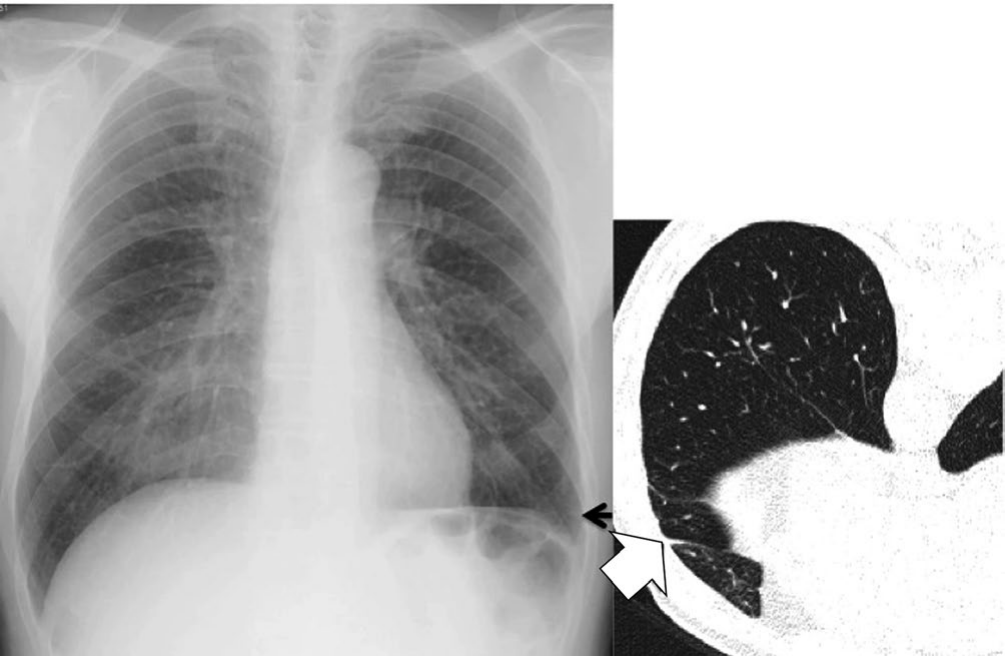
Discrepancy between chest radiography and thin‐slice CT reading results (Case 2). Old pleurisy misclassified as pleural plaque by a NIOSH B reader and an AIR Pneumo reader (black arrow). Another AIR Pneumo reader correctly classified this as diffuse pleural thickening, pleural abnormalities continued from cost‐phrenic angle obliteration. As the costophrenic angle obliteration is very subtle on chest radiography, it will be very difficult to detect CT‐proven pleurisy (white arrow)

**FIGURE 4:**
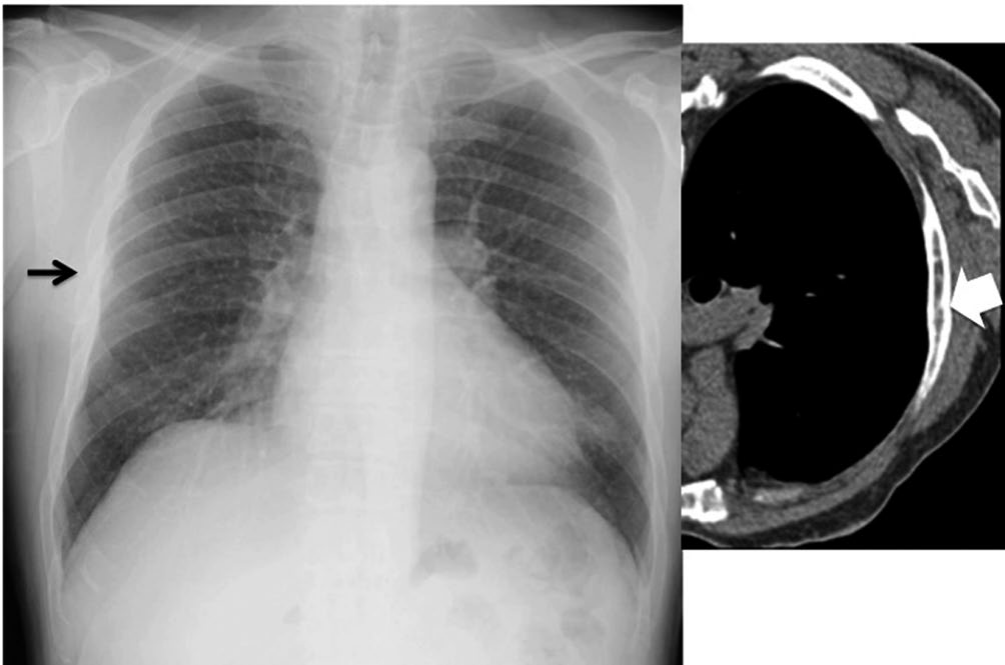
Discrepancy between chest radiography and thin‐slice CT reading results (Case 3). CT‐proven fractured rib (white arrow) misclassified as pleural plaques by three AIR Pneumo readers. A NIOSH B reader correctly classified this (black arrow) as fractured rib

Theoretically, radiography is, of course, inferior to CT in detecting subtle findings including tiny parenchymal abnormalities^21^, ^22^ and pleural plaques mainly owing to unavailability of cross‐sectional images that can be easily acquired on thin‐slice CT (Figure 2). Most moderate pleural changes were found on the lateral chest wall and on the diaphragm; however, many small plaques in these regions registering on thin‐slice CT were difficult to detect on chest radiographies as expected. Not only false negative cases but also false positive cases were found on chest radiography. Pleuritis or rib fractures in some cases were falsely diagnosed as pleural plaques on chest radiographies (Figures 3 and 4). Face‐on calcified plaques and emphysematous changes were also possible causes of false positive cases for irregular/linear opacities. Despite its known disadvantage compared to chest CT, chest radiography is affordable in most clinical settings and shows a sensitivity of 55.6% (5/9) for parenchymal lesions and 36.4% (16/44) for pleural lesions by reader A, respectively, even in construction workers with mild exposure to asbestos, suggesting its utility as a screening and/or surveillance test for employees.

Emphysematous changes found in our study population might be related to dust exposure including asbestos^23^; however, the possibility of a relationship with habitual smoking has yet to be examined in our study. In silicosis and coal worker's pneumoconiosis, progressive massive fibrosis causes distortion of the lung which consequently damage peripheral lungs to cause secondary emphysema. Focal emphysema is formed in p‐type pneumoconiosis seen in coal dust exposure by increasing air space size.^8^, ^24^ Dust‐related emphysematous changes are reported in cases with exposure to metals,^25^ including indium compounds, the latter have reports of characteristic progressive emphysema shown in follow‐up studies.^26^, ^27^, ^28^

There was a positive trend between sensitivities, especially when 0/1 was used as a cut‐off, and years of experience in interpretation of pneumoconioses on chest radiographies, suggesting the importance of years of experience for improvement of diagnostic performance and proper application of the training system for occupational health physicians. The physician trained through the AIR Pneumo program with 3 years' experience demonstrated a competency equivalent to the NIOSH B reader in diagnostic performance, resulting in no significant difference in sensitivity and specificity for both parenchymal and pleural abnormalities.

There are some limitations to this study. First, the small number of positive cases in our study population introduces statistical limitations in determining the significant differences in sensitivity for the parenchymal abnormalities, underscoring the need for further studies with a larger number of subjects. Second, the possibility of selection bias exists because willing participants in the study population might have had a higher prevalence of abnormalities than the general population of construction workers. Third, the number of physicians who interpreted chest radiographies was relatively small and may not reflect the ability of physicians in a general clinical setting.

In conclusion, thin‐slice CT‐detected irregular opacity was found in 9.3%, whereas pleural plaque was found in 45.4% among the construction workers. Chest radiography showed acceptable performance in classifying pneumoconiotic opacities according to ILO/ICRP 2000 by the AIR Pneumo and/or NIOSH‐certified physicians. There was a positive trend between sensitivities and experience in interpretation of pneumoconiosis on radiographies. The reading results of AIR Pneumo‐certified readers were almost comparable to that of a NIOSH B reader.

## DISCLOSURE

*Approval of the research protocol*: The ethical committee of the University of Fukui approved this study, No. 97 on July 26th, 2005. *Informed consent:* Informed consent for chest radiography and CT was acquired by all the enrolled subjects. *Registry and the registration no. of the study/trial:* N/A. *Animal studies:* N/A.

## CONFLICT OF INTEREST

The authors declare that there are no conflict of interest.

## AUTHOR CONTRIBUTION

SN and NS conceived the ideas; SN, MN, NAJ‐P, TM, NNR, TT, YK, and HI collected the data; SN, MN, and SN analyzed the data; SN, MN, NAJ‐P, and NS contributed to the writing of the manuscript.
